# ASL demonstrates higher and more homogenous calf muscle perfusion with post-occlusion hyperemia than with exercise

**DOI:** 10.1186/1532-429X-15-S1-P216

**Published:** 2013-01-30

**Authors:** David Lopez, Amy Pollak, Craig H Meyer, Ronny Jiji, Frederick H Epstein, Jennifer R Hunter, John M Christopher, Christopher M Kramer

**Affiliations:** 1 Medicine, University of Virginia, Charlottesville, VA, USA; 2 Radiology, University of Virginia, Charlottesville, VA, USA; 3 Biomedical Engineering, University of Virginia, Charlottesville, VA, USA; 4 Cardiovascular Imaging Center, University of Virginia, Charlottesville, VA, USA

## Background

Pulsed arterial spin labeling (PASL) is a non-contrast MRI technique capable of quantifying calf muscle perfusion with comparable accuracy to contrast enhanced-MRI perfusion in normal subjects and peripheral arterial disease (PAD) patients. Peak perfusion can be achieved with exercise or during post-occlusion hyperemia. However, exercise stress may underestimate peak perfusion because of submaximal effort and/or heterogeneous flow. We hypothesized that post-occlusion hyperemia would yield higher peak calf muscle flow compared to peak exercise calf muscle flow measured with PASL.

## Methods

Twenty-five normal subjects with no PAD risk factors or exertional symptoms were enrolled. Peak calf muscle perfusion was measured in 15 volunteers at peak exercise (Ex-PASL) and in 10 volunteers during post-cuff occlusion hyperemia (Cuff-PASL). Ex-PASL volunteers performed supine plantar flexion exercise using a pedal ergometer until exhaustion. Cuff-PASL volunteers had a thigh cuff inflated to 200 mmHg for 5 minutes. At end-exercise or immediately after cuff deflation, 15 averaged PASL images were acquired using a single-shot echo-planar pulse sequence (Total scan time 62s, FOV 200x200 mm, matrix 64x64, TR 4000 ms, TE 32 ms, 10 mm thick). PASL was performed using the proximal inversion with control for off-resonance effects technique and proximal blood labeling. The QUIPSS II with thin-slice TI1 periodic saturation technique minimized errors from variable transit delay of spins from labeling region to imaging slice and contamination of perfusion signal by intravascular blood. Peak perfusion was measured by placing a region of interest in the calf muscle group with the greatest signal intensity.

## Results

**Figure 1:**
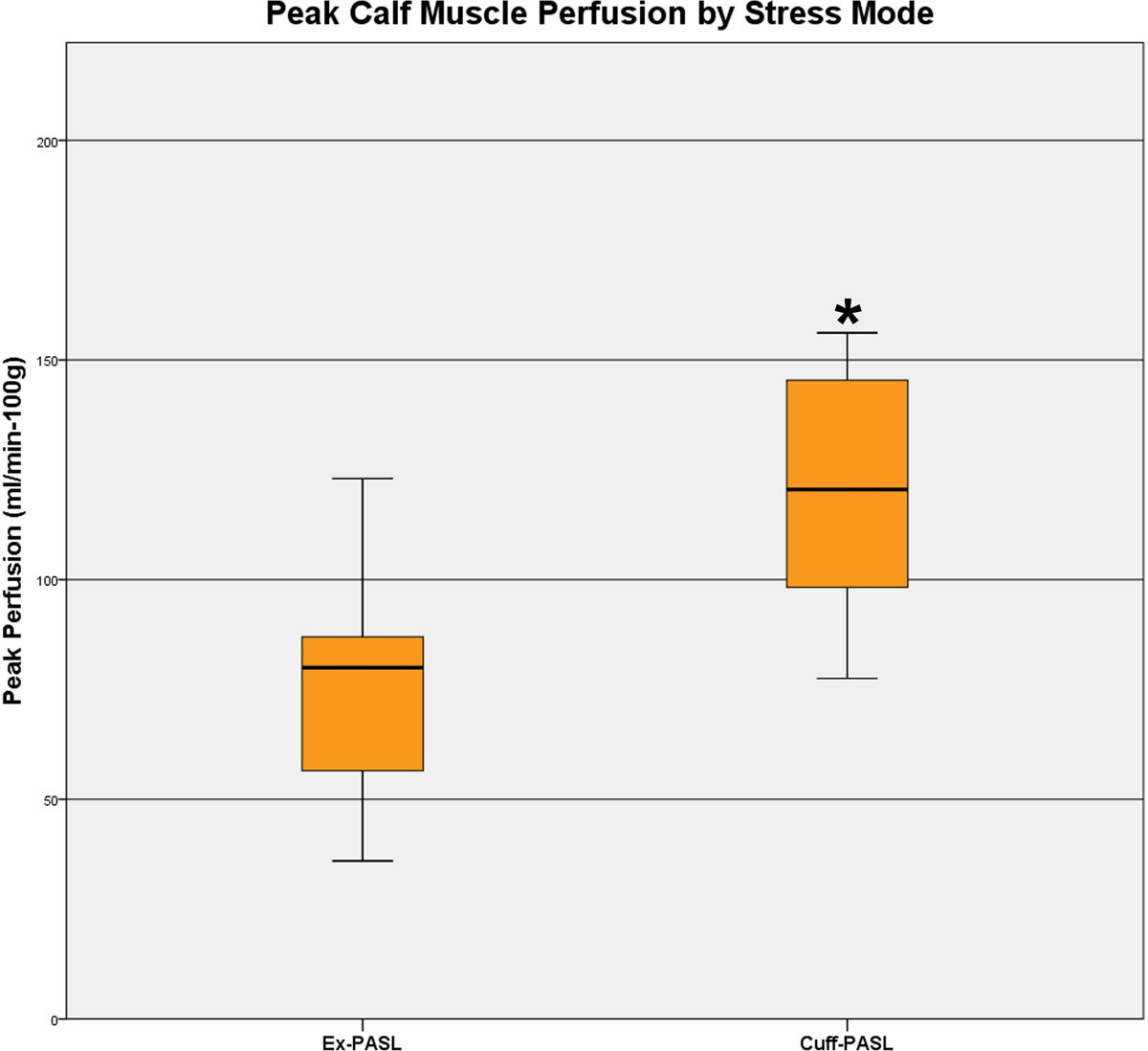
Box plot of peak calf muscle perfusion in Ex-PASL and Cuff-PASL subjects.* p<0.0001 for Cuff-PASL compared to Ex-PASL.

**Figure 2:**
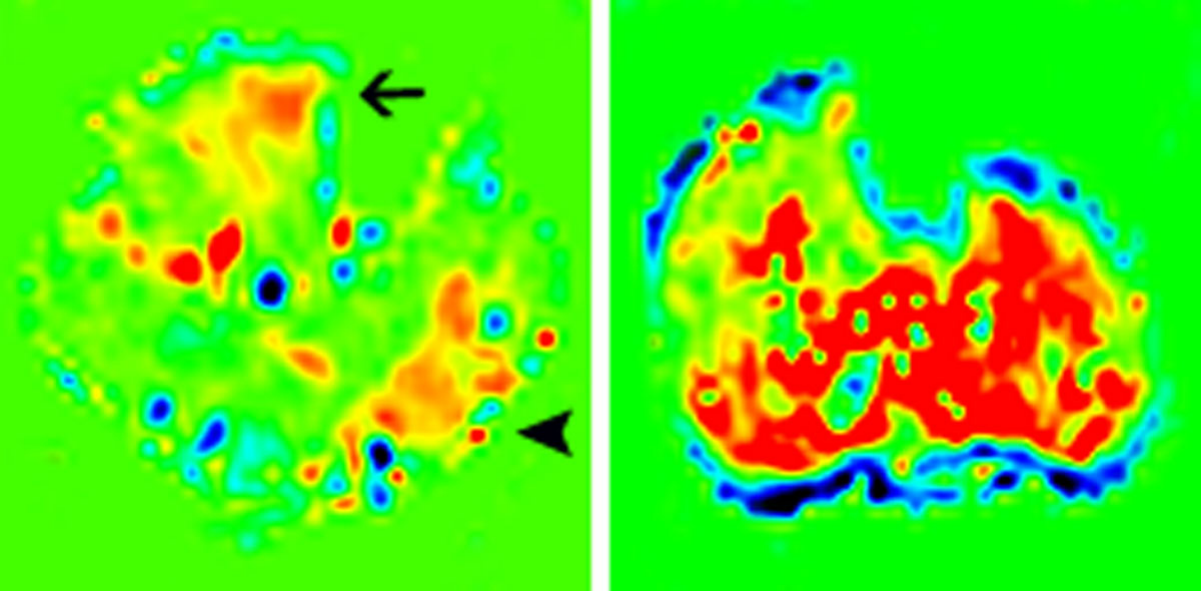
Flow distribution in Ex-PASL (A) compared to Cuff-PASL (B). At peak exercise flow is greater in the anterior tibialis (arrow) and gastrocnemius (arrow head). During post-cuff occlusion hyperemia flow is more homogenously increased throughout all muscle groups.

Fifteen of the subjects had ABI testing and all values were normal. Mean age was 54±9 years for Ex-PASL and 54±7 years for Cuff-PASL. Exercise time (mean±SD) was 631±388s. Peak calf muscle perfusion (mean±SD) was higher with Cuff-PASL than with Ex-PASL (121±29 mL/min-100g vs. 77±23 mL/min-100g, p<0.0001), mean difference 44 mL/min-100g (95% CI 22-65mL/min-100g). (Figure 1) Compared to Ex-PASL, Cuff-PASL hyperemia yielded a more homogeneous leg perfusion. (Figure 2)

## Conclusions

Post-cuff occlusion hyperemia yields higher and more homogeneous peak calf muscle perfusion compared to peak exercise in normal volunteers. These characteristics make cuff occlusion an attractive form of stress for patients with PAD in whom peak perfusion may be underestimated due to exercise limiting symptoms. However, exercise does allow testing of specific muscle groups used in the exercise and is a more physiologic stress. Further studies are needed to compare the performance and reproducibility of the two techniques in PAD patients.

## Funding

R01 HL075792, 5T32EB003841

